# Draft genome sequences of four plant-associated free-living diazotrophs isolated from the rhizosphere of Malian tomato

**DOI:** 10.1128/mra.00568-24

**Published:** 2024-10-22

**Authors:** Gabriele Bellotti, Paul Sanou, Piero Sunzini, Vincenzo Tabaglio, Pier Sandro Cocconcelli, Edoardo Puglisi

**Affiliations:** 1 Department for Sustainable Food Process, Università Cattolica del Sacro Cuore, Piacenza, Italy; 2 Tamat E. T. S., Perugia, Italy; 3 Department of Sustainable Crop Production, Università Cattolica del Sacro Cuore, Piacenza, Italy; University of California Riverside, Riverside, California, USA

**Keywords:** PGPR, biostimulants, rhizosphere, diazotrophs

## Abstract

This announcement reports the draft genome sequences of two *Azospirillum argentinense* and two *Azotobacter salinestris* isolated from the rhizosphere of a tomato plant grown in a village in the Republic of Mali. These strains are plant growth-promoting rhizobacteria and are highly valuable to agriculture for their ability to fix atmospheric nitrogen.

## ANNOUNCEMENT

*Azospirillum* and *Azotobacter* belong to the class of *Alphaproteobacteria* and comprise spiral (spirillum) or curved rod (vibrioid) cells, and oval (coccus) or rod-shaped (bacillus) cells, respectively. They are diazotrophs, meaning that they can perform biological nitrogen fixation where atmospheric nitrogen (N_₂_) is converted into forms available for living organisms. Despite being considered “free-living” diazotrophs, these two genera are often found in close contact with plant roots. Indeed, several studies have shown their abilities to shape plant root architecture and exert several other plant growth-promoting activities such as phytohormone production (1, 2).

**Fig 1:**
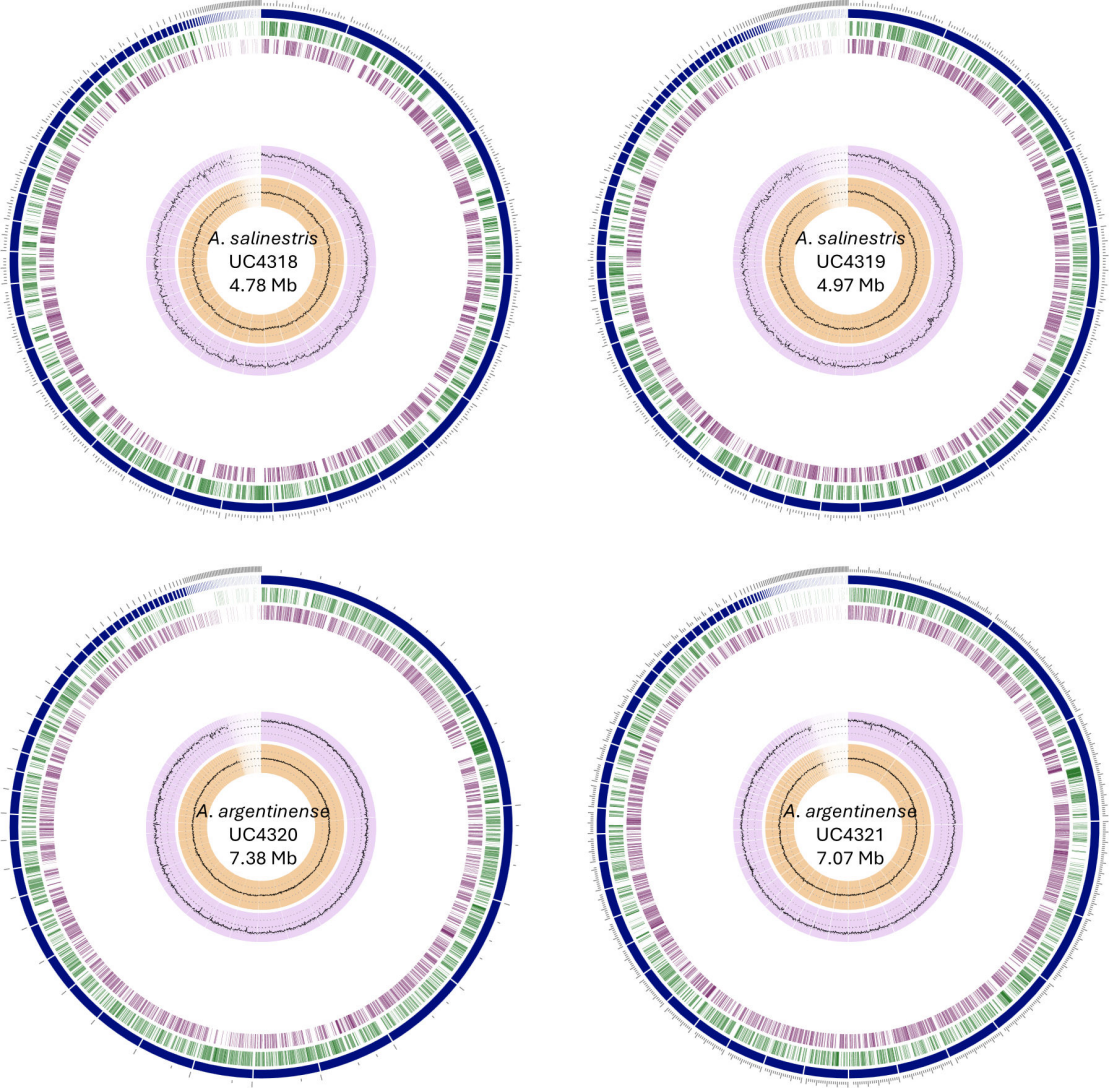
Circular graphical display of the distribution of the genome features in annotated genomes. From outer to inner rings: the contigs, coding sequence (CDS) on the forward strand, CDS on the reverse strand, GC content, and GC skew estimated from the annotation pipeline on www.bv-brc.org.

In this announcement, the draft genomes of two strains of *Azotobacter salinestris* and two *Azospirillum argentinense* isolated from the rhizosphere of a tomato plant are reported. Plant roots were collected in the village of N'Piébougou, in the Koulikoro region of the Republic of Mali, during the dry season in February 2023. The rhizosphere was obtained with single-use sterile tweezers. Bacterial isolation was performed on nitrogen-free malate agar plates (3) preceded by an enrichment technique, with incubation in shaking conditions at 30°C for 48 h. Serial dilutions were plated on nitrogen-free malate agar plates and incubated at 30°C for 3–4 days. The resulting colonies were streaked out on Luria-Bertani (LB) three times to obtain axenic cultures. For long-term preservation, cells were harvested in 7% DMSO LB and kept at −45°C. Genomic DNA was obtained from cells cultured in 10 mL tubes containing 3 mL of Tryptic Soy Broth (Madrid, Spain) and incubated by shaking at 30°C for 24 h. DNA was extracted from 1 mL cultures using the E.Z.N.A. Bacterial DNA Kit (Omega Bio-tek, Georgia, USA) following the manufacturer’s instructions. Raw reads were obtained on a platform NovaSeq 6000 at Novogene (Cambridge, UK) using Illumina Technology PE150. The genomic DNA was randomly sheared into short fragments. The obtained fragments were end repaired, A-tailed, and ligated with Illumina adapters. Fragments were PCR amplified, size selected, and purified. The library was checked with Qubit and real-time PCR for quantification and bioanalyzer for size distribution detection and quality checked. Novogene’s QC report revealed that raw reads had an average Phred score of 35–38. Reads were submitted to BV-BRC’s assembly service (www.bv-brc.org) for assembly and annotation (4) Default parameters were used except where otherwise noted. Reads were assembled using the Unicycler version 0.4.8 (5). The draft genome obtained was then annotated using the RAST tool kit (RASTtk) (6). The total number of raw reads, depth, and other relevant statistics on the assembly and annotation were made with QUAST version 5.2.0, EvalG, and EvalCon tools on the BV-BRC pipeline (7). The results are reported in Table 1. A circular graphical display of the genome annotations is provided in Fig. 1. All sequenced strains harbor the genes encoding the nitrogenase enzyme complex, the Fe protein:MoFe protein complex (*nifDK*), and the ATP-dependent electron-donating Fe-protein (*nifH*), required for the reduction of atmospheric nitrogen to ammonia.

**TABLE 1:** List of bacterial isolates, summary of genome features, accession numbers, and relevant statistics of raw sequences and assembled genomes of *Azotobacter salinestris* strains UC4318, UC4319 and *Azospirillum argentinense* strains UC4320, UC4321

Taxonomy			Raw reads				Assembly								Annotation								
Strain	Species	ANI % vs type strain	Accession biosample ID	SRA accession no.	No. of raw reads (bp)	GC %	Genome accession ID	Assembly size (Mbp)	Average depth (short reads)	Average short read coverage	No. of contigs	GC %	*N*_50_	*L*_50_	Complete-ness (%)	Coarse consistency (%)	Fine consistency (%)	Contamination (%)	Total number of coding sequences	Protein-encoding genes with functional assignment	Protein-encoding genes without functional assignment	Duplicated genes	Underpresent genes
UC4318	*Azotobacter salinestris*	95.9514	SAMN41552033	SRR29195087	18,179,350	65.09	JBDZCZ000000000	4.775631	565.46456	565.464	87	66.04	178,766	11	93.5	98.4	96.1	4.3	4,831	3,020	1,689	59	76
UC4319	*Azotobacter salinestris*	95.9009	SAMN41552034	SRR29195086	17,625,360	64.91	JBDZDA000000000	4.975393	527.11261	527.112	118	65.89	148,865	10	93.6	98.5	96.2	4.6	5,069	3,116	1,836	57	75
UC4320	*Azospirillum argentinense*	98.4212	SAMN41552035	SRR29195085	17,292,764	67.85	JBDZDB000000000	7.385184	348.21683	348.217	97	68.69	340,004	7	100	95.9	90.4	0.5	7,071	3,822	3,070	99	32
UC4321	*Azospirillum argentinense*	95.2057	SAMN41552036	SRR29195084	18,423,740	68.31	JBDZDC000000000	7.078652	386.60078	386.601	106	69.06	256,309	8	99.9	95.3	90.6	0.3	6,698	3,633	2,918	93	37

## Data Availability

This Whole Genome Shotgun project named “Free living diazotrophs genome sequencing” has been deposited on NCBI under the BioProject accession PRJNA1116845. Raw reads and assemblies are available under the accession numbers reported in Table 1.
